# On-Demand Release of Drug from Magnetic Nanoparticle-Loaded Alginate Beads

**DOI:** 10.1155/2021/5576283

**Published:** 2021-04-02

**Authors:** Dung The Nguyen, Nguyet-Minh Nguyen, Duc-Minh Vu, Minh-Duc Tran, Van-Thao Ta

**Affiliations:** ^1^Faculty of Chemistry, VNU University of Science, Vietnam National University, Hanoi, 19 Le Thanh Tong, Hoan Kiem, Hanoi 11021, Vietnam; ^2^Nguyen Tat Thanh High School, Hanoi National University of Education, 136 Xuan Thuy, Cau Giay, Hanoi 11310, Vietnam; ^3^Department of Medical Engineering, Hanoi Medical University, 1 Ton That Tung, Dong Da, Hanoi 116177, Vietnam

## Abstract

Targeted delivery and controlled release of drugs has been considered to be an important therapeutic approach since it could allow a better treatment efficiency and less side effects. In this research, magnetite Fe_3_O_4_ nanoparticles were successfully synthesized via the coprecipitation method and then loaded in alginate beads with berberine as a drug model for drug release application. Various factors such as pH values of the suspended environment and surface modifications of the drug carrier could be exploited to adjust the amount of drug release. More importantly, the amount of drug release could be effectively controlled by an on-off switching operation of a static magnetic field.

## 1. Introduction

Magnetic nanoparticles are of great interest because of their action-at-a-distance behavior with an applied magnetic field. Important capabilities of magnetic nanoparticles are the external controllability of magnetic forces. Utilization of magnetic field for biomedical applications including contrast agent, hyperthermia, and drug delivery has been bursting since the last decade due to the development of concepts and tools derived from nanotechnology [1–3]. New paradigms for theranostics have been created by developing multifunctional systems combining therapeutic and diagnostic agents within a single platform [4–6]. As a part of this, numerous research studies have been conducted with attempts to exploit significant advantages of delivery and controlled release of drug molecules, including the ability to target specific locations in the body and the reduction of the quantity of drug needed for better treatment efficiency with minimum severe side effects. It has been reported and reviewed that a magnetostatic field was able to accumulate magnetic nanoparticles at a targeted location even for a deep target, and thus, drug molecules which attached onto the magnetic nanoparticles could be successfully delivered [7, 8].

Once the carriers containing magnetic nanoparticles and drug molecules were concentrated at the target, the drug molecules were then released by various factors including changes of the local physicochemical environment and application of an external magnetic field. For example, it was reported that a decrease in the pH of release media caused a pronounced increase in polymer degradation as well as drug release [9–12]. The magnetic-triggered drug release by an alternating current field have been strongly investigated since the alternating current field caused heat generation from the magnetic nanoparticles which could be utilized to mechanically deform the polymer matrix or to open the gates of drug carrier [13–15]. An alternating current field could activate a combination thermos-chemotherapy as synergistic effects. Several studies reported that by using an alternative field to raise temperature to 42°C, the drug would burst out while cancer cell viability decreased [16, 17]. It was also reported that the heat generationactivated temperature-sensitive cation channels within cells in order to enhance drug uptake without observable toxic effects [18].

Functionalization of the magnetic nanoparticles required careful considerations on the choices of polymer matrix carrier and the interactions or linkages with the drug molecules in consideration with physicochemical stability, targeting ability, drug loading, and release. Alginate and alginate-based materials have been extensively used in the biomedical field, including tissue engineering, wound dressings, drug delivery, dental impressions, transplantation, encapsulation, cell therapy, and so on [19]. So far, alginates have been ranked as one of the most abundantly used biopolymers. A nanocomposite hydrogel containing alginate and mesoporous silica exhibited a drug loading efficacy of 48%–90% with a steady release rate [20]. In another report, Yoncheva et al. [21] encapsulated doxorubicin in chitosan/alginate nanoparticles by electrostatic binding of the drug in order to explore the enhancement of therapeutic response. Hydrophobic and dipole-dipole interactions were recommended as crucial factors to optimize the loading efficacy and to promote the sustained release of drug.

In this study, magnetic nanoparticles and alginate were synthesized and then loaded into alginate beads with berberine as a drug model. The characteristics of the magnetic nanoparticles and the drug and magnetic nanoparticle-loaded alginate beads were investigated thoroughly. The drug and magnetic nanoparticle-loaded alginate beads were used for controlled release of drug molecules under an external magnetic stimulation. Instead of exploiting benefits of an alternative current field, we found that an on-demand drug release via the on-off operation of a static magnetic field could offer a mechanism to minimize drug release during delivery process and to maintain drug level at reasonable levels within the desired therapeutic range for a long time.

## 2. Materials and Methods

### 2.1. Preparation of Magnetic Nanoparticles

The magnetic nanoparticles were synthesized using a simple chemical coprecipitation method as described previously [22]. Typically, 10 mL of an alkali solution at 95°C was added into 100 mL solution of Fe precursor at the same temperature which contains FeCl_3_ (Sigma-Aldrich, ≥98%) and FeCl_2_ (Sigma-Aldrich, ≥99%) with a molar ratio of 2 : 1 and FeCl_2_ concentration of 10^−3^ M. The solution was kept heating and rapidly stirring for 1 hr. The resultant black dispersion of the magnetic nanoparticles was then collected, washed, and dried in vacuum. The magnetic nanoparticles were characterized by Powder XRD patterns using a Philips X'Pert PRO MPD X-ray diffractometer. The particle morphology was observed by a TEM image taken with a JEOL JEM-2011 transmission electron microscope. The magnetic properties were measured by using a Lake Shore 7300 vibrating sample magnetometer.

### 2.2. Preparation of Magnetic Nanoparticle-Loaded Alginate Beads

The cross-linked network of the alginate beads has been formed by strong ionic bonds of alginate with calcium ions. In brief, 50 mL solution containing 0.5 gram of berberine (Sigma-Aldrich) and 0.5 gram of the magnetic nanoparticles synthesized before was prepared. To this, 0.5 gram of sodium alginate (Sigma-Aldrich) was added under gentle stirring to ensure complete dissolution. After that, the solution was added dropwise using a 0.5 *μ*L syringe to CaCl_2_ (Junsei, ≥95%) solution of a definite concentration (0.5% (m/v), 1.0% (m/v), and 2.0% (m/v)). The beads were then collected and dried in vacuum.

In order to investigate effects of alginate beads surface modification on the drug release, the drug and magnetic nanoparticle-loaded alginate beads were gently dipped into a solution of gelatine 1.0% (m/v) or chitosan 1.0% (m/v) for short time before being collected and dried in vacuum.

### 2.3. Drug Release Investigation

The preweighed amount of drug and magnetic nanoparticle-loaded alginate beads (approximately 0.5 gram) was placed in 50 mL of release medium (acetate buffer solution pH = 4 or phosphate buffer solution pH = 7) with a gentle shake. After regular time intervals, 5 mL of the release medium was withdrawn and its absorbance was measured spectrophotometrically at 348 nm to determine the berberine concentration [23]. The same amount of corresponding buffer solution was added to the release medium to replace the taken amount for measurement of berberine concentration. In order to stimulate an on-demand controlled release of drug molecules by an external static magnetic field, a permanent magnet of 3.5 Tesla was applied during monitoring the drug release profile. Two specific conditions were designed. In the first condition, the static magnetic field was applied for 45 minutes and then was removed. In the second one, the static magnetic field was applied for the whole time. The quantity of drug released was calculated using Lambert–Beer's plot obtained for drug solutions of known concentrations. The cumulative fraction release of berberine was calculated after normalizing the cumulative release after time *t* (*M*_*t*_) with the cumulative release at time infinity (*M*_*∞*_).

## 3. Results and Discussion

### 3.1. Characterization of Magnetic Nanoparticles

**Figure 1:**
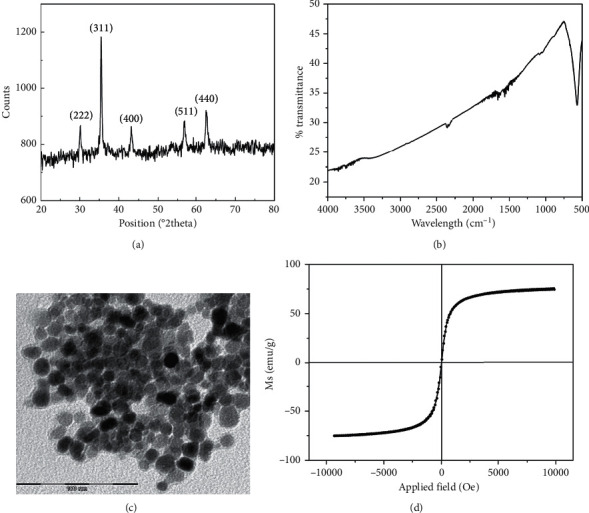
Characterization of the magnetic nanoparticles prepared by the coprecipitation method. (a) X-ray diffraction pattern. (b) FT-IR spectrum. (c) TEM measurement. (d) VSM analysis.

Characteristics of the magnetic nanoparticles prepared by the coprecipitation method were investigated thoroughly. As indicated in Figure 1(a), a very well-formed crystalline structure corresponding to the (222), (311), (400), (511), and (440) planes with a lattice parameter of *a* = 8.3778 Å was obtained. All XRD peaks corresponded to the characteristic peaks of magnetite Fe_3_O_4_ crystal without any presence of other iron oxide forms (reference code: 01-071-6336). As shown in Figure 1(b), a typical peak of Fe-O lattice mode of Fe_3_O_4_ appeared at nearly 584 cm^−1^ [24]. No other peak was clearly determined, confirming that the magnetite Fe_3_O_4_ nanoparticles were synthesized without any surface modification. The morphology of the typical Fe_3_O_4_ nanoparticles was observed as shown in Figure 1(c). Since the magnetic nanoparticles were prepared without presence of any surfactant, the agglomeration might happen inside solution due to the hydrophobic property or it could have occurred during drying of the sample on the TEM grid to reduce surface energy of those small-size nanoparticles. However, it could be clearly seen that a large quantity of uniform, quasi-spherical particles with an average primary diameter of about 10 nm was synthesized. As a critical result of small-size effects, the magnetite nanoparticles exhibited superparamagnetic properties with a magnetization saturation of 73 emu/g and negligible remanence as shown in Figure 1(d).

### 3.2. Characterization of Drug and Magnetic Nanoparticle-Loaded Alginate Beads

**Figure 2:**
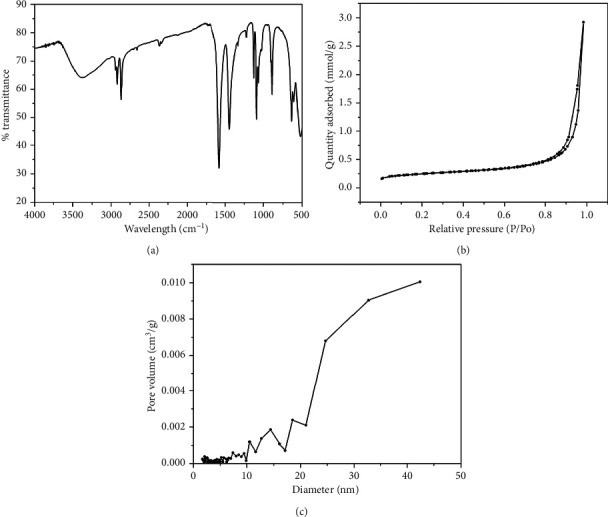
(a) FT-IR spectra of the magnetic nanoparticles (Fe_3_O_4_ NPs) and the magnetic nanoparticle-loaded alginate beads. (b) The adsorption-desorption isotherm and pore size distribution (inset) of the magnetic nanoparticle-loaded alginate beads.

Figure 2(a) exhibited the FT-IR spectra of the drug and magnetic nanoparticle-loaded alginate beads. It could be clearly seen that peaks at 1576 cm^−1^, 1445 cm^−1^, and 1089 cm^−1^ which corresponded to the asymmetric and symmetric stretching of the -COO^−^ groups and C-O stretching of the -COO^−^ groups [25] were clearly identified for the magnetic nanoparticle-loaded alginate beads. It should be mentioned that when the mixture containing sodium alginate solution and other materials such as the magnetic nanoparticles and drug molecules was dropped into a solution of CaCl_2_, the Ca^2+^ ions instantaneously reacted with the -COO^−^ groups and cross-linked the alginate chains, hardened the outer layer of alginate to form beads, and simultaneously wrapped up other materials including the magnetic nanoparticles and drug molecules inside [19, 26].

Figures 2(b) and 2(c) exhibit the nitrogen adsorption-desorption isotherm and pore size distribution of the drug and magnetic nanoparticle-loaded alginate beads. The beads showed pore in the range of few nanometers to few tenth nanometers with a average pore size of about 20 nm. The porosity structure helped the beads to absorb solvent to swell and eventually allowed trapped drug molecules to diffuse out of the beads.

### 3.3. Drug Release from the Magnetic Nanoparticle-Loaded Alginate Beads

**Figure 3:**
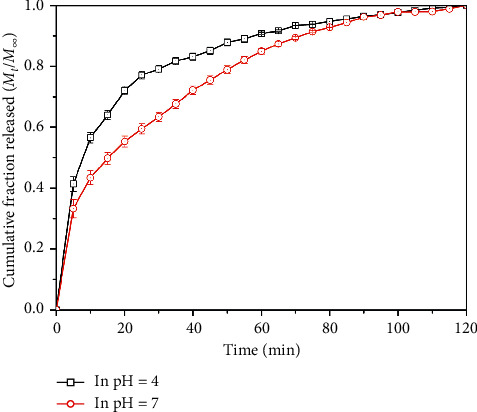
Cumulative fraction released profile of drug molecules in the acetate buffer solution (pH = 4) and in the phosphate buffer solution (pH = 7).

The release of berberine drug molecules from the alginate beads was investigated in different media as indicated in Figure 3. It could be clearly observed that the drug molecule was released faster in the acetate buffer solution (pH = 4) than in the phosphate buffer solution (pH = 7). About 80% of the cumulative release of drug was obtained in the acetate buffer solution after 25 min while that amount in the phosphate buffer solution was about 55% after similar time interval. Most drug molecules were released after 60 min in the acetate buffer solution, while it had to be at least 100 min for most drug molecules to be released in the phosphate buffer solution.

The faster release of the drug molecules in the acetate buffer solution could be attributed to the effect of the H^+^ ion in the solution. In the acetate buffer solution, because of higher H^+^ concentration, the H^+^ ions could weaken the binding between the alginate polymer strands via reaction with Ca^2+^ ions, thus caused the beads to swell more significantly and degrading the polymer layer which results in the faster diffusion of the drug molecules from the beads to the surroundings. Owing to that difference in the microenvironment, a change in biological pH occurred by disease would result in polymer degradation enough for significant difference of drug release from the carriers [27, 28].

**Figure 4:**
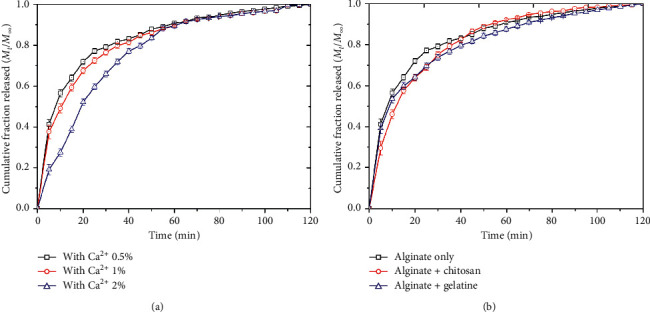
Cumulative fraction released profiles of drug molecules in the acetate buffer solution (pH = 4) from the beads prepared (a) in different Ca^2+^ concentration solutions and (b) with surface modification with chitosan and gelatine.

In order to control the drug release from the alginate beads, several factors were investigated such as varying the Ca^2+^ concentration when generating the beads or by shortly dipping the beads into the chitosan or gelatine solutions for surface modification. By increasing the Ca^2+^ concentration when generating the beads, it would be expected that more Ca^2+^ ions would react with the -COO^−^ groups and cross-link the alginate chains tighter. As a result, the drug molecules which were trapped inside the beads would be diffused out harder as indicated in Figure 4(a). In case of surface modification of the beads, chitosan and gelatine have been used widely with alginate to form composite for drug delivery and controlled release. Chitosan is a cationic biopolymer obtained from the deacetylation of chitin, carrying unbranched chains of (1,4)2-acetoamido-2-deoxy-D-glucose, and gelatine is a mixture of peptides and proteins produced by partial hydrolysis of collagen extracted from animals. Modification of alginate with chitosan and gelatin could be prepared under simple and mild reaction conditions in water by electrostatic gelation of oppositely charged interactions as widely reported [29, 30]. Since chitosan and gelatine would be easily adsorbed onto the alginate beads, the chitosan and gelatine would form another layer on the alginate beads' surface, thus causing the slower drug release as indicated in Figure 4(b). However, it should be emphasized that the effects of those factors on the release of the drug molecules from the beads were not significantly obtained.

**Figure 5:**
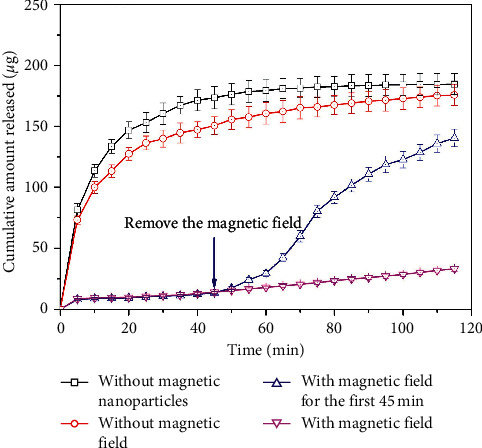
Cumulative released profile of drug molecules with and without trigger of a static magnetic field.

The release profiles of the drug molecules from the drug and magnetic nanoparticle-loaded alginate beads with effects of a static magnetic field were investigated as shown in Figure 5. It was clearly observed that without the magnetic nanoparticles as well as without an applied field, the drug molecule release profile was similar to above investigations. Drug release from such polymeric matrices generally was based on either by solute diffusion and/or polymer dissolution. By applying a static magnetic field, the drug release rate significantly decreased. As time increased, the alginate structure became swollen even though the static magnetic field was maintained. The drug molecules, therefore, could diffuse out slightly faster but an accumulated amount of the drug in the media was far lower than without application of the static magnetic field. This observation supported that the static magnetic field could preserve the drug molecules during delivery as well as maintain the drug release at low concentration at targeted place.

In order to obtain an on-demand release profile of the drug, the static magnetic field was applied for the first 45 min and then removed. Within the first 45 min with a strong effect of the static magnetic field, the amount of the drug released from the beads was maintained low. However, by removing the static magnetic field, the beads become swollen as usual and thus induce the release of the drug molecules from inside the beads to the surroundings.

Without the presence of the static magnetic field, the magnetic nanoparticles inside the alginate beads were subjected to zero magnetization due to their superparamagnetic properties as characterized and thus the drug release profile exhibited a normal diffusion mode. When applying the static magnetic field, the magnetic moments of the nanoparticles tended to align with the magnetic field which induced the magnetic nanoparticles to aggregate instantly, leading to a rapid decrease in the porosity of the beads. Therefore, the drugs molecules were restrictedly confined within the network of the alginate beads, causing a significant reduction in the diffusion of the drug to the release medium. This preliminary investigation suggested that a controlled release model with a predetermined released amount of the drug could be designed by operating “on-off” cycles of a given magnetic field.

## 4. Conclusions

Magnetic nanoparticles were successfully synthesized and loaded into the alginate beads with berberine as the drug model for controlled drug release application. The magnetic nanoparticles of average diameter of 10 nm were prepared with superparamagnetic properties. The magnetic nanoparticle-loaded alginate beads were simply formed by reaction of the alginate with Ca^2+^ which helped to wrap up the nanoparticles and drug molecules inside the beads. The amount of drug release could be controlled by designing an external on-off operation of a given magnetic field. This observation suggested that the controlled release of drug with an adjustable amount can be properly performed for practical needs with an external magnetic control of the magnetic nanoparticle-loaded beads for controlled delivery of therapeutic drugs.

## Data Availability

The data used to support the findings of this study are included within the article.
